# Improvements in sleep quality and fatigue are associated with improvements in functional recovery following hospitalization in older adults

**DOI:** 10.3389/frsle.2022.1011930

**Published:** 2022-10-14

**Authors:** Emily J. Arentson-Lantz, Rachel R. Deer, Manasa Kokonda, Chelsey L. Wen, Thomas A. Pecha, Samantha A. Carreon, Trung M. Ngyen, Elena Volpi, Sara Nowakowski

**Affiliations:** 1 Department of Nutrition, Metabolism, and Rehabilitation Sciences, University of Texas Medical Branch, Galveston, TX, United States,; 2 Sealy Center on Aging, University of Texas Medical Branch, Galveston, TX, United States,; 3 Center for Innovation in Quality, Effectiveness, and Safety, Michael DeBakey VA Medical Center, Houston, TX, United States,; 4 Department of Medicine, Baylor College of Medicine, Houston, TX, United States,; 5 School of Public Health, University of Texas Health Science Center, Houston, TX, United States,; 6 School of Medicine, Baylor College of Medicine, Houston, TX, United States,; 7 Department of Pediatrics, Baylor College of Medicine, Houston, TX, United States,; 8 Department of Internal Medicine, University of Texas Medical Branch, Galveston, TX, United States

**Keywords:** sleep quality, fatigue, hospitalization, physical function, independence

## Abstract

**Study objectives:**

Poor sleep quality, a frequent problem in older adults, has been shown to be associated with reduced physical function and wellbeing. However, little is known about the relationship between sleep quality and the recovery of physical function following hospitalization. Thus, we conducted this study to examine the association between sleep quality and functional recovery after an acute hospitalization in community dwelling older adults.

**Methods:**

Older adult patients (*N* = 23, mean age = 74 ± 9 years) were recruited during an acute hospitalization (average length of stay 3.9 days) with a cardiovascular (56%), pulmonary (22%), or metabolic (13%) admission diagnosis. Objective physical function was measured using the Short Physical Performance Battery (SPPB) and self-reported function was assessed with Katz Index of Independence in Activities of Daily Living (ADL) and Lawton Instrumental Activities of Daily Living Scale (IADL). Sleep quality was measured using Pittsburgh Sleep Quality Index (PSQI) global score and Iowa Fatigue Score (IFS). Testing was performed prior to discharge (baseline) and 4-weeks post-discharge (follow-up).

**Results:**

Regression models showed PSQI Subjective Sleep Quality change scores from baseline to 4-week follow-up predicted a change in ADL (β = −0.22); PSQI Use of Sleep Medications change scores predicted a change in SPPB Total (β = 1.62) and SPPB Chair Stand (β = 0.63); IFS change scores predicted SPPB Total (β = −0.16) and SPPB Chair Stand performance (β = −0.07) change scores.

**Conclusions:**

For older adults, changes in sleep medication use, daytime dysfunction, and fatigue were associated with improvements in functional recovery (including physical performance and independence) from acute hospitalization to 4-week follow-up. These results suggest that interventions focused on improving sleep quality, daytime consequences, and fatigue might help enhance physical functioning following hospitalization.

**Clinical trial registration:**

ClinicalTrials.gov, identifier: NCT02203656.

## Introduction

Older adults commonly suffer from multiple chronic conditions that can lead to hospitalization. From 2000 to 2015, more than 3 out of 10 adults in the US were admitted to an acute care hospital, and in 2018, the average length stay of older adults was 5.6 days (Weiss and Elixhauser, 2014; Quality AfHR, 2021). Additionally, older adults are also more likely to be physically incapacitated or placed on bed rest for extended periods (Boyd et al., 2005; Brown et al., 2009). Compared to younger adults on bed rest, older adults on bed rest experience significantly more functional decline, muscle loss, and accumulation of inflammatory cells in skeletal muscle (Tanner et al., 2015; Reidy et al., 2018). Physical inactivity alone contributes to a host of negative outcomes including impaired insulin action, loss of lean muscle mass, fatigue, impaired functional capacity, and increased morbidity and mortality (English et al., 2016; Arentson-Lantz et al., 2019, 2020; Wang et al., 2020). Reductions in physical function and activities of daily living are commonly observed in older patients admitted for acute hospital care (Greysen, 2016; Hartley et al., 2020). These impairments linked to hospitalization, collectively termed ‘hospital deconditioning’, are often still present at discharge, and many patients fail to fully recover to pre-hospitalization physical function levels (Salpakoski et al., 2014; Morisawa et al., 2021). Hospital deconditioning has been identified as an emerging risk factor of increased frailty, disability and dependence that contributes to re-hospitalization, reduced quality of life, and mortality (Keim et al., 2020; Saitoh et al., 2021).

Identifying factors that may be related to physical functioning is a key step in understanding recovery and facilitating independence following hospital discharge. Indeed, adequate sleep is considered a necessary prerequisite to healing and recovery from illness, and sleep disturbances can hinder postoperative recovery (Su and Wang, 2018). However, 45% of hospitalized older adults report poor disturbed sleep, with an average of 3.75 h total sleep time and 13 awakenings per night (Blackwell et al., 2014; Stone et al., 2014). While data suggest that changes in sleep are associated with changes in physical functioning (Chennaoui et al., 2014), sleep studies in the hospital have largely been limited to measures of subjective sleep in critical care. Previous research has demonstrated that improvements in sleep quality are associated with better exercise function (Chennaoui et al., 2014; Antunes et al., 2017), while poorer sleep quality is assciated with worse mobility and physical function (Vitale et al., 2019), especially among older adults (Campanini et al., 2019). Sleep quality is a subjective rating of sleep based on a person’s perception of sleep. Sleep quality is defined as rating the ability to fall and stay asleep, ability to return to sleep after an awakening, sleep duration, and whether or not an individual feels refreshed or restored in the morning (Harvey et al., 2008; Yilmaz et al., 2017). Conversely, increased mobility and exercise have been linked to better sleep quality (Chennaoui et al., 2014; Rubio-Arias et al., 2017) and shorter sleep latency (Passos et al., 2011), while reduced mobility has been shown to lead to sleep disturbance (Kirkhus et al., 2019). Given this reciprocal relationship, studies examining changes in sleep quality following hospital discharge and its relationship to changes in functional recovery are greatly needed.

Thus, we evaluated the relationship between sleep quality as assessed by Pittsburgh Sleep Quality Index (PSQI) and physical functioning as assessed by Short Physical Performance Battery (SPPB) in older adults during their hospital stay and at a 4-week follow-up. As secondary outcomes, we examined the relationship of fatigue as well as some self-reported measures of physical function and independence with the primary outcomes, PSQI and SPPB. We hypothesized that improvements in sleep quality and fatigue would be associated with improvements in physical performance following hospital discharge.

## Methods

Participants, aged 65 years and older, were recruited and written consent was obtained during an acute hospitalization to the Acute Care for Elders Unit at the University of Texas Medical Branch (UTMB) hospital in Galveston, TX. All participants were from a larger parent study designed to identify optimal strategies to accelerate functional recovery from hospitalization examine the feasibility of enrollment and assessment of hospitalized older adults (Deer et al., 2016, 2018). Briefly, feasibility was assessed through collection of the number of eligible, contacted, and enrolled patients; reasons for declining enrollment and study withdrawal; and adherence with completing study procedures. The patients were assessed in the hospital and at 1-week and 4-week post-discharge. Each assessment measured muscle strength, physical function/performance, body composition, and psychological function. Physical activity levels were continuously monitored throughout study participation. This protocol was approved by the UTMB Institutional Review Board. This study was registered with ClinicalTrials.gov as NCT02203656.

A total of 100 participants were randomized in the parent study; however, collection of sleep assessment data was initiated later in the data collection period of the parent study, so we were able to collect sleep/fatigue related outcomes from 23 participants. Potential participants were identified by daily chart review and were approached about participation in the study during their hospital stay. The inclusion for this present study and the parent study were identical. To be eligible for inclusion in the parent study, participants had to meet the following general criteria: (i) admitted to the hospital with an acute onset of disease or condition; (ii) residing at home before and after hospitalization; (iii) able to consent to participate in the study; (iv) self-reported ability to walk across a small room (with or without an assistive device) 2 weeks prior to hospitalization; (v) ability to stand independently at baseline testing; (vi) no medical contraindication to wearing the loose fitting velcro strap of a step activity monitor on one ankle; (vii) living within 30 miles of the hospital; and (viii) aged 65 years or older. If a patient’s planned discharge location changed after enrollment from home to placement into skilled nursing or rehabilitation facility, the participant was withdrawn from the study.

Exclusion criteria were: (i) nursing home resident or hospice patient; (ii) uncontrolled blood pressure (systolic > 150, or diastolic > 100 mmHg); (iii) history of stroke with motor disability; (iv) estimated glomerular filtration rate < 30 mL/min/1.73 m2 or evidence of kidney failure; (v) liver disease (AST/ALT 2 times above the normal limit, hyperbilirubinemia); (vi) recent (within 3 months) treatment with anabolic steroids; (vii) planned/elective hospitalization within 30 days of discharge; (viii) cognitive dysfunction determined by chart review, reported by nursing staff, or observed by trained research staff (not alert or oriented, dementia, active delirium); (ix) living >30 miles from the hospital; and (x) any other condition or event considered exclusionary by the principal investigator or treating physician.

Briefly, all participants in this present study completed the following sleep, fatigue and physical function related questionnaires as well as an objective measure of physical function at baseline (during the hospital stay). On average, the participants completed the baseline assessment 1.9 days into their hospital stay. The same measures (unless otherwise noted below) were repeated at 4-week follow-up study visit (27–33 days post-discharge) performed in the participant’s home or at the UTMB facility. The age-adjusted Charleston Comorbidity Index score (Charlson et al., 1987) was calculated at baseline as a description of underlying comorbidities.

### Sleep assessment

The Pittsburgh Sleep Quality Index (PSQI) is a 19-item validated survey instrument designed to assess general sleep quality (Buysse et al., 1989). Seven clinically derived components scores are summed to yield one global score (ranging from 0 to 21), with high scores indicating poorer general sleep quality. The component scores include Subjective Sleep Quality, Sleep Latency, Sleep Duration, Habitual Sleep Efficiency, Sleep Disturbances, Use of Sleeping Medications, and Daytime Dysfunction. A Global PSQI score of ≥ 5 indicates clinically significant poor sleep quality. In this present report, we reported both the global and component scores as others have found that a summed global score does not capture the multidimensional nature of sleep disturbance in older adults (Cole et al., 2006). Sleep Environment Questionnaire (SEQ) is a 10-question questionnaire that was modified to assess how the hospital environment impacted their sleep (see Table 3). This questionnaire was adapted in-house from the Assessment of Sleep Environment questionnaire (Olivier et al., 2016) and was only collected at baseline for descriptive analysis. All questions were reformatted as yes/no questions and specifically asked about the hospital environment impact on sleep. The SEQ was only intended to be descriptive for quality improvement purposes and responses reported as a percentage of participants answered ‘yes’ to the question. A version of the original SEQ and modified SEQ are available in Supplementary Figure 1.

### Fatigue assessment

Iowa Fatigue Scale (IFS) is an 11-question survey designed to assess fatigue in terms of cognitive function, drowsiness, energy level, and productivity, with a higher score indicating greater fatigue (Hartz et al., 2003). Scores range from 11 to 55, with scores of 30–39 indicative of fatigue and scores of 40–55 indicative of severe fatigue.

### Physical performance assessment

All participants completed the following physical function-related testing and questionnaires at baseline before the participant was discharged from the hospital and at 4-week follow-up. The Short Physical Performance Battery (SPPB) is an objective measure of physical performance in older adults and consists of a total score as well as three component scores of lower body function: a short, timed walk at usual gait speed (SPPB Gait), five repeated chair stands (SPPB Chair Stand), and a standing balance exercise (SPPB Balance) (Guralnik et al., 1994). Each of the three performance measures were scored from 0 to 4, with 0 indicating inability to complete the test and 4 indicating the highest level of performance. A one point change in the total SPPB score is considered to be clinically meaningful (Perera et al., 2006). We also used the Katz Index of Independence in Activities of Daily Living (ADL), a 6-item questionnaire which assesses independence in bathing, dressing, toileting, transferring, continence, and feeding (Katz, 1983). Each item is scored as 1- independent or 0- dependent. Higher total scores indicate greater levels of independence. The Lawton Instrumental Activities of Daily Living Scale (IADL) is an 8-item questionnaire of independent living skills containing with a summary score ranging from 0 (low function) to 8 (high function) (Lawton and Brody, 1969). Items include: using the telephone, shopping, preparing food, housekeeping, doing laundry, using transportation, handling medications, and handling finances. Higher scores indicate a higher level of independent functioning.

### Statistical analyses

A paired sample *t*-test was performed to detect the significant difference between baseline and 4-week follow-up testing. Correlation analysis was performed between the change from baseline to follow-up in sleep measures and the change in physical function measures. Covariates were selected based on documented association with sleep and/or physical functioning based on prior published studies and the parent study. A team of content experts reviewed and selected the final list of variables to assess their association with PSQI and/or SPPB in the present study. Covariates were selected for inclusion in the stepwise model based upon their association with the outcome at *p* < 0.05. Age, sex, race, ethnicity, hospital admission diagnosis, hospital length of stay, and Charlson Comorbidty Index were all considered as covariates but did not meet *p* < 0.05 criteria to be included in the model. Components that significantly correlated with the outcome variable were considered for inclusion in the multiple regression model. Outcome variables were assessed for normality and homogeneity tests to satisfy the distribution assumptions. Stepwise regression method was used to find the best fit regression model. Multivariable regression model was used for continuous outcome variables (SPPB measures, ADL, IADL) and a logistic regression model was used for analyzing categorical outcome variables. Significance was set at 0.05, and *p*-values were adjusted by Bonferroni procedure when conducting multiple comparisons. All analyses were performed using SAS software version 9.4 (SAS Institute, Inc., North Carolina, USA).

## Results

### Demographics

The average age of participants in this study (*n* = 23) was 74.4 ± 9.0 years old with a BMI of 29.7 ± 5.7 (Table 1). The majority were female (82.6%), white (82.6%), and non-Hispanic (87.0%). The average length of stay for acute hospitalization was 3.9 days, with Cardiovascular (56.5%), Pulmonary (30.4%), or Metabolic (13.0%) as the discharge diagnosis. The average age-adjusted Charleston Comorbidity Index score was 5.5.

### Sleep and physical performance scores at baseline and 4-week follow-up

**FIGURE 1:**
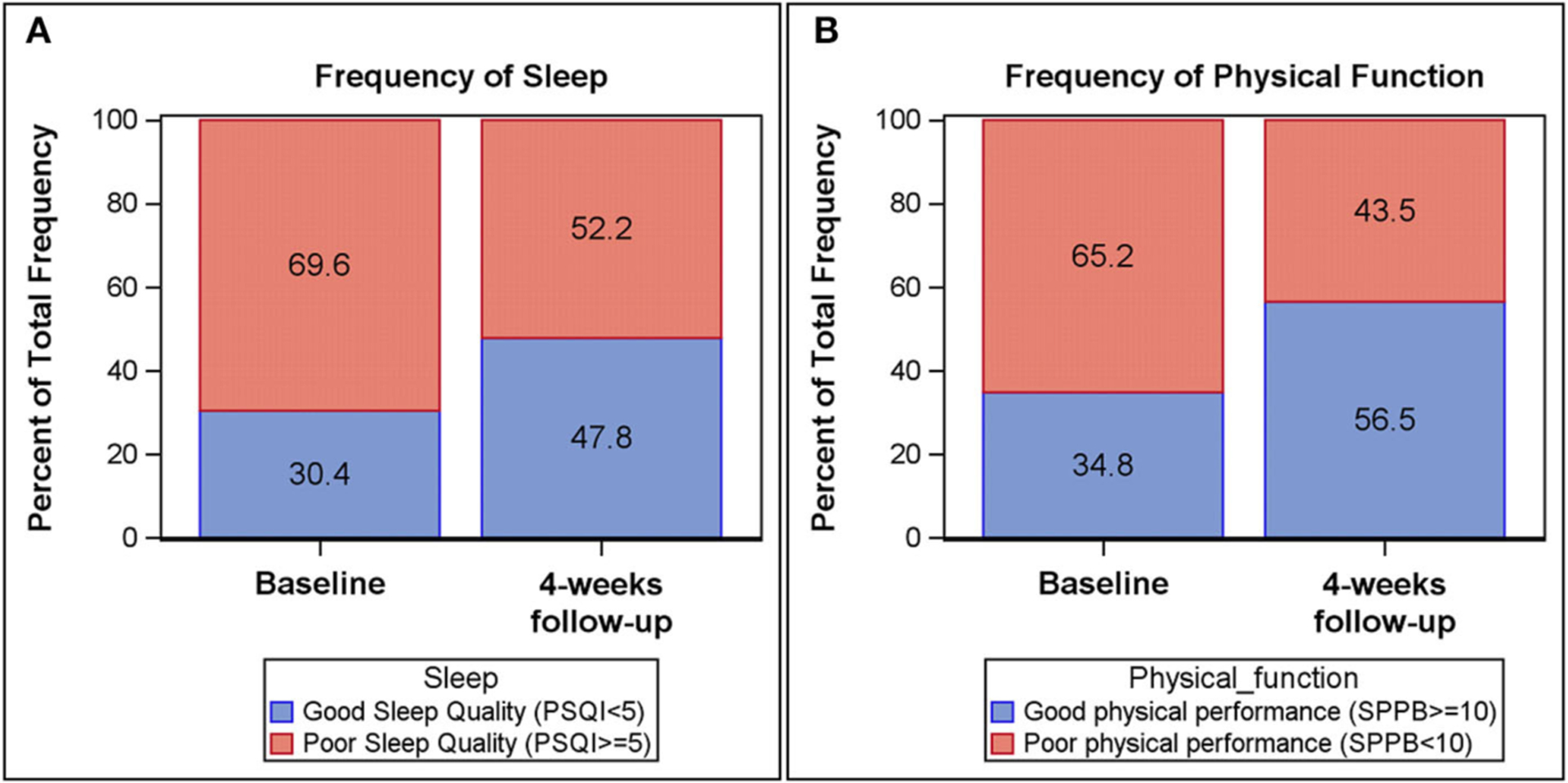
Frequency plots of Pittsburgh Sleep Quality Index (PSQI) global score and Short Physical Performance Battery (SPPB) total score at baseline and 4-week follow-up. Good sleep quality (PSQI < 5) among participants is increased from baseline (30.4%) to 4-week follow-up (47.8%) **(A)**. Likewise, physical performance (SPPB > 10) among participants increased from baseline (34.8%) to 4-week follow-up (56.5%) **(B)**.

Self-reported measures of sleep and daytime fatigue, as measured by the PSQI and IFS, improved longitudinally from baseline to follow-up (Table 2 and Figure 1). The Global PSQI score decreased 4 weeks post hospital discharge (*p* = 0.13), with a significant decrease in sleep disturbance (*p* = 0.03). At baseline, 69.6% of participants reported poor sleep quality (Global PSQI ≥ 5) while 52.2% reported poor sleep quality at 4-week follow-up. Participants also reported a lower level of fatigue 4 weeks post-hospital discharge (IFS decreased by 3 units, *p* = 0.13). Physical function improved (i.e., increase in scores) from baseline to follow-up across both global and components measures of the SPPB. Both the SPPB Total score (*p* = 0.03) and SPPB Balance score (*p* = 0.03) were significantly improved at 4-week follow-up. At baseline 65.2% of participants demonstrated poor physical performance (SPPB Total <10) while 43.5% demonstrated poor physical performance at follow-up.

### Hospital sleep environment

The majority of participants (76.4%) stated that their sleep during hospitalization differed from their usual sleep patterns at home (Table 3). Common issues included: being uncomfortable or pain disturbing sleep (41.2%), uncomfortable mattress or pillows (29.4%), and engaging in other behaviors that disturbed sleep like watching TV (23.6%).

### Impact of change in sleep quality on change physical function recovery

To determine if the trajectory of physical function recovery after hospitalization was impacted by changes in sleep, we examined the association in change in sleep quality and physical function from baseline to 4-week follow-up (Table 4). Our results demonstrated improvements in sleep positively impacted the trajectory of functional recovery improvements. The PSQI Sleep Quality component score was inversely associated with an increase in ADL score, −0.22 ± 0.09, *p* = 0.03; indicating improved sleep quality predicted higher independence. PSQI Daytime Dysfunction component score was inversely associated with the change score of SPPB Balance, −0.51 ± 0.19, *p* = 0.02; indicating improved daytime dysfunction predicted improved physical performance. The change score of PSQI Use of Sleep Medications component score was significantly associated with increased SPPB Total score, 1.62 ± 0.45, *p* = 0.002 and SPPB Chair Stand score, 0.63 ± 0.22, *p* = 0.01; indicating greater use of sleep medications predicted improved physical performance. Similarly, the IFS change scores were inversely associated with physical performance (SPPB Total score, −0.16 ± 0.06, *p* = 0.01 and SPPB Chair Stand score, −0.07 ± 0.03, *p* = 0.03) and independence (increased IADL score, −0.11 ± 0.04, *p* = 0.005). Indicating reduced fatigue predicted improvements in physical performance and higher independence. Non-significant associations are listed in Table 4. Generalized regression plots of the significant associations are found in Supplementary Figure 2.

## Discussion

The present study evaluated the relationship between sleep quality and physical performance among a sample of hospitalized older adults. Of note, patients in this sample were not admitted to intensive care and were acutely hospitalized for an average length of stay of 3.9 days. Improvements in self-reported sleep quality following hospital discharge predicted improvements in objective measures of physical functioning following hospitalization. Specifically, we found that decreased report of daytime consequences of poor sleep predicted improvement in balance. Moreover, self-reported improvement in fatigue and increase in sleep medication use predicted improvements in some aspects of lower extremity physical performance. These results suggest that, regardless of poor sleep upon admission to the hospital, interventions targeting sleep quality, daytime consequences and fatigue may enhance functional recovery following hospital discharge and increase levels of independence, particularly in older patients that are more prone to deleterious effects of extended bedrest and immobility.

Our results are consistent with prior studies that link poor sleep quality with worse mobility and physical function (Chennaoui et al., 2014; Antunes et al., 2017) especially among older adults (Campanini et al., 2019). A strength of our study is the longitudinal design that allows us to examine Subjective sleep quality, fatigue, physical performance, and independence during the acute hospital stay and again 4 weeks following hospital discharge. Of note, during our initial analysis, we found age was not a significant covariate with change SPPB Total score and its component scores; this may be due to the small sample size or a result of differing discharge diagnoses. Although we did not find an association between sleep quality and physical functioning during the hospital stay (Supplementary Table 1), we did find that changes in sleep quality (i.e., improvements in daytime functioning) and improvements in self-reported fatigue were associated with improvements in physical functioning from hospital stay to 4-week post-hospital discharge. These results are in line with a study examining subjective meaning of sleep quality. Investigators found that both healthy sleepers and patients with insomnia considered feeling rested and restored on waking and alert throughout the day as among the most important factors for judging sleep quality (Akhavan et al., 2010). The consequences of poor sleep quality include decreased daytime alertness (Katz, 1983; Guralnik et al., 1994), attention/concentration (Orff et al., 2007; Fortier-Brochu and Morin, 2014), motivation (Soehner and Harvey, 2012), and physical activity along with increased fatigue and compensatory behaviors (e.g., napping) (Gooneratne et al., 2003; Chasens et al., 2007; Foley et al., 2007; Alessi et al., 2008). These daytime sequelae may serve to undermine recovery from acute hospitalization. This, in turn, may explain increased disability, nursing home placement (Martin et al., 2010; Fung et al., 2012), morbidity (Foley et al., 2004), and mortality (Manabe et al., 2000; Dew et al., 2003; Cappuccio et al., 2010) in older adults with sleep problems. Targeted interventions focusing on improving sleep quality and reducing daytime consequences of poor sleep may be beneficial for hospitalized older adults.

It should be noted that the sleep medication use component score of PSQI was associated with improved physical performance in this study. Current treatment options to improve sleep in the hospital setting are limited and typically include pharmacotherapy. Acceptability of sedative hypnotics, however, is limited due to increased risk of falls (Diem et al., 2014), prolonged length of stay, and higher costs in the acute hospital setting (Zisselman et al., 1996; Yuen et al., 1997). Although over the conter sleep medications such as diphenhydramine, doxylamine as well as prescription sleep medications such as zolpidem and eszopiclone have been shown to be effective in the treatment of insomnia, they are not recommended for older adults due to concerns about tolerance, abuse, dependence, and rebound insomnia following discontinuation (Bloom et al., 2009; Bourgeois et al., 2013; Abraham et al., 2017). Thus, these limitations and concerns establish an imminent need for non-pharmacological approaches to ameliorate sleep disturbances for this population during hospital admissions and post-discharge (Kripke et al., 1998).

There are several limitations to our study that should be noted when evaluating the results from this study. First, examining sleep quality was an ancillary aim to the overall study that examined the effect of hospitalization on physical functioning. We were limited to collecting sleep data from 23 participants with complete data during and following hospitalization. While the small sample size limits generalizability, this is an important first step to acquire preliminary data, which was meant to be hypothesis generating. Secondly, our assessment of sleep quality using the PSQI was conducted on average 1.9 days after hospital admission. Standard PSQI instructions include to assess sleep habits over the past month with answers most accurate for the majority of days and nights in the past month. The PSQI administration is subject to memory/recall bias. Participants may have placed more emphasis on sleep during the hospital due to saliency and recency bias. In addition, we did not inquire about past history of sleep quality or sleep concerns, and therefore cannot make statements comparing prior episodes vs. new onset of sleep disturbances during hospitalization; we also cannot speak to the impact of duration of sleep disturbances in relation to study outcomes. Finally, the observational nature of our design meant that we cannot make any assertions about directionality or causality, including any causal role of poor sleep on physical recovery observed here.

Future work should address the limitations of the present study. To strengthen these findings, longitudinal studies, beyond 4 weeks post-discharge, could test the possibility that persistent sleep problems may have more robust effects on physical functioning. Assessment of insomnia history vs. new onset should also be assessed. Randomized clinical trials targeting sleep during and following hospitalization would allow us to test if sleep is a potential modifiable risk factor or intervention target to improve functional recovery in hospitalized older adults.

In summary, improvements in self-reported sleep (specifically daytime functioning) and fatigue were associated with improvements in physical functioning and independence pre-post hospitalization. These findings challenge the assumption in the lay community that sleep problems in older adults are solely a troublesome symptom of aging to be tolerated, with little implication for health and recovery. Future work should consider the benefits of treating sleep problems in older adults to facilitate functional recovery following hospital discharge.

## Supplementary Material

Supplementary Figure 1

Supplementary Table 1

Supplementary Figure 2

## Figures and Tables

**TABLE 1:** Demographics and hospitalization characteristics of study cohort^a^.

Variable	
Age; mean (SD)	74.4 (9.0)
BMI; mean (SD)	29.7 (5.7)
ACCI; mean (SD)	5.5 (1.3)
Sex; F/M	19/4
Race; *n* (%)	
Black or African American	4 (17.4)
White	19 (82.6)
Ethnicity; *n* (%)	
Hispanic	3 (13.0)
Non-Hispanic	20 (87.0)

a Sample size = 23, values presented as mean (SD) or *n* (%).

SD, standard deviation; BMI, body mass index; ACCI, Age-Adjusted Charleston Comorbidity Index.

**TABLE 2:** Sleep and physical performance scores at baseline, at 4-week follow-up and their change scores^a^.

Variable	Baseline	4−week follow−up	Estimated Mean Difference	*p*−value^b^
PSQI total	6.4 (4.3)	4.7 (3.1)	−1.69 (3.64)	0.14
PSQI sleep quality	0.96 (0.71)	0.69 (0.55)	−0.26 (0.75)	0.17
PSQI sleep latency	1.22 (1.04)	1.08 (1.20)	−0.13 (1.01)	0.69
PSQI sleep duration	0.43 (1.04)	0.26 (0.75)	−0.17 (1.03)	0.52
PSQI sleep efficiency	0.74 (1.09)	0.57 (0.89)	−0.17 (1.11)	0.56
PSQI sleep disturbance	1.34 (0.64)	0.92 (0.67)	−0.43 (0.59)	**0.03** *
PSQI use of sleep medication	0.91 (1.24)	0.69 (1.25)	−0.22 (1.04)	0.56
PSQI daytime dysfunction	0.83 (1.12)	0.52 (0.89)	−0.31 (1.33)	0.31
IFS	28.2 (5.77)	25.2 (7.13)	−3.0 (7.86)	0.13
SPPB total	6.78 (4.26)	9.26 (3.02)	2.42 (2.75)	**0.03** *
SPPB balance	2.52 (1.48)	3.39 (1.08)	0.87 (1.36)	**0.03** *
SPPB gait	2.39 (1.55)	3.08 (1.12)	0.67 (1.09)	0.09
SPPB chair stand	1.87 (1.77)	2.78 (1.54)	0.87 (1.26)	0.07
ADLS	5.74 (0.62)	5.79 (0.59)	−0.37 (0.88)	0.81
IADLS	8.35 (1.78)	8.39 (2.13)	0.04 (1.27)	0.94

* Indicates significant p-value < 0.05.

a Values are reported as means with standard deviation.

b *p*-values calculated with chi-square test for categorical variables and paired t-test for continuous variables.

PSQI, Pittsburgh Sleep Quality Index; IFS, Iowa Fatigue Score; SPPB, Short Physical Performance Battery; ADLS, Activities of Daily Living Score; IADLS, Instrumental Activities of Daily Living Score.

The bold values are significant (*p* < 0.05).

**TABLE 3:** Descriptive statistics of Sleep Environment Questionnaire (SEQ) participants completed prior to discharge form the hospital^a^.

Question	True *n* (%)
My sleep differed during my time at UTMB hospital than my usual sleep patterns at home	13 (76.4)
The room was so warm or cold that it disturbed my sleep	3 (17.6)
Light from the window or other source disturbed my sleep	1 (5.9)
The mattress or pillow(s) were uncomfortable and disturbed my sleep	5 (29.4)
During the night, I could hear noise outside the room that disturbed my sleep	0 (0.0)
Noise inside the room (from the air vents, pipes, etc.,) was so loud that I was aware of the sounds and it disturbed my sleep	4 (23.6)
The concern and support of staff provided me a level of comfort such that I rarely if ever worried about being safe at night	17 (100)
I engaged in any behaviors during a time I would normally be asleep at home (e.g., watched TV, read, worried, worked, checked email/internet)	4 (23.6)
It was uncomfortable (or caused me pain) being confined to bed and this disturbs my sleep	7 (41.2)
Other things related to being at the UTMB hospital disturbed my sleep	3 (17.6)

a Of the 23 participants, only 17 completed this questionnaire.

**TABLE 4:** Linear regression analysis between the change in measures of sleep and fatigue (PSQI and IFS) and change in physical function between baseline and 4-week follow-up^a^.

Variable	Change SPPB total	Change SPPB balance	Change SPPB Chair-stand	Change SPPB gait	Change ADL	Change IADL
Change PSQI total	0.01 (0.16)	−0.01 (0.08)	0.05 (0.07)	−0.03 (0.06)	−0.03 (0.02)	−0.06 (0.07)
Change PSQI sleep quality	1.11 (0.93)	0.13 (0.56)	0.58 (0.45)	0.38 (0.44)	−0.22 (0.09)*	0.29 (0.49)
Change PSQI sleep latency	−0.03 (0.50)	−0.03 (0.31)	0.06 (0.24)	−0.06 (0.24)	−0.09 (0.06)	0.01 (0.26)
Change PSQI sleep duration	−1.27 (0.79)	−0.17 (0.47)	−0.52 (0.39)	−0.57 (0.38)	−0.29 (0.11)	−0.10 (0.41)
Change PSQI sleep efficiency	1.68 (0.81)	0.55 (0.48)	0.55 (0.40)	0.57 (0.38)	0.32 (0.11)	−0.06 (0.43)
Change PSQI sleep disturbance	−0.31 (0.96)	−0.31 (0.57)	0.16 (0.47)	−0.16 (0.46)	0.02 (0.13)	**1.04 (0.46)** *
Change PSQI use of sleep medications	**1.62 (0.45)** *	0.49 (0.33)	**0.63 (0.22)** *	0.57 (0.26)	0.11 (0.07)	−0.31 (0.29)
Change PSQI daytime dysfunction	−0.37 (0.42)	−0.51 (0.19)*	0.30 (0.20)	−0.18 (0.20)	−0.02 (0.05)	0.25 (0.22)
Change IFS	−0.16 (0.06)*	−0.02 (0.04)	−0.07 (0.03)*	−0.05 (0.03)	−0.02 (0.01)	−0.11 (0.04)*

a Values are represented as β (SE). β = regression coefficient (represents the slope of the linear relation of the predictor variable and the outcome variable), SE, standard error.

* Indicates significant *p*-value < 0.05.

PSQI, Pittsburgh Sleep Quality Index; SPPB, Short Physical Performance Battery; ADL, Activities of Daily Living Score; IADL, Instrumental Activities of Daily Living Score.

The bold values are significant (p < 0.05).

## Data Availability

The raw data supporting the conclusions of this article will be made available by the authors, without undue reservation.

## Abbreviations:

PSQI: Pittsburgh Sleep Quality Index
IFS: Iowa Fatigue Score
SPPB: Short Physical Performance Battery
ADL: Activities of Daily Living
IADL: Instrumental Activities of Daily Living
SEQ: Sleep Environment Questionnaire
BMI: body mass index
ACCI: age-adjusted Charleston comorbidity index

## References

[R1] Abraham O, Schleiden L, and Albert SM (2017). Over-the-counter medications containing diphenhydramine and doxylamine used by older adults to improve sleep. Int. J. Clin. Pharm 39, 808–817. doi: 10.1007/s11096-017-0467-x 28466395 PMC5541127

[R2] Akhavan T, Luhovyy BL, Brown PH, Cho CE, and Anderson GH (2010). Effect of premeal consumption of whey protein and its hydrolysate on food intake and postmeal glycemia and insulin responses in young adults. Am. J. Clin. Nutr 91, 966–975. doi: 10.3945/ajcn.2009.28406 20164320

[R3] Alessi CA, Martin JL, Webber AP, Alam T, Littner MR, Harker JO, (2008). More daytime sleeping predicts less functional recovery among older people undergoing inpatient post-acute rehabilitation. Sleep 31, 1291–1300. doi: 10.5665/sleep/31.9.1291 18788654 PMC2542969

[R4] Antunes BM, Campos EZ, Parmezzani SS, Santos RV, Franchini E, Lira FS, (2017). Sleep quality and duration are associated with performance in maximal incremental test. Physiol, Behav. 177, 252–256. doi: 10.1016/j.physbeh.2017.05.014 28502838

[R5] Arentson-Lantz E, Galvan E, Wacher A, Fry CS, and Paddon-Jones D (2019). 2,000 steps/day does not fully protect skeletal muscle health in older adults during bed rest. J Aging Phys Act 27, 191–197. doi: 10.1123/japa.2018-0093 29989486 PMC6710835

[R6] Arentson-Lantz EJ, Fiebig KN, Anderson-Catania KJ, Deer RR, Wacher A, Fry CS, (2020). Countering disuse atrophy in older adults with low volume leucine supplementation. J. Appl. Physiol 128, 967–977. doi: 10.1152/japplphysiol.00847.2019 32191600 PMC7191508

[R7] Blackwell T, Yaffe K, Laffan A, Ancoli-Israel S, Redline S, Ensrud KE, (2014). Associations of objectively and subjectively measured sleep quality with subsequent cognitive decline in older community-dwelling men: the MrOS sleep study. Sleep 37, 655–663. doi: 10.5665/sleep.3562 24899757 PMC4044750

[R8] Bloom HG, Ahmed I, Alessi CA, Buysse DJ, Ancoli-Israel S, Kryger MH, (2009). Evidence-based recommendations for the assessment and management of sleep disorders in older persons. J. Am. Geriatr. Soc 57, 761–789. doi: 10.1111/j.1532-5415.2009.02220.x 19484833 PMC2748127

[R9] Bourgeois J, Elseviers MM, Van Bortel L, Petrovic M, and Vander Stichele RH (2013). Sleep quality of benzodiazepine users in nursing homes: a comparative study with nonusers. Sleep Med. 14, 614–621. doi: 10.1016/j.sleep.2013.03.012 23692988

[R10] Boyd CM, Xue QL, Guralnik JM, and Fried LP (2005). Hospitalization and development of dependence in activities of daily living in a cohort of disabled older women: the Women’s Health and Aging Study I. J. Gerontol. A Biol. Sci. Med. Sci 60, 888–893. doi: 10.1093/gerona/60.7.888 16079213

[R11] Brown CJ, Redden DT, Flood KL, and Allman RM (2009). The underrecognized epidemic of low mobility during hospitalization of older adults. J. Am. Geriatr. Soc 57, 1660–1665. doi: 10.1111/j.1532-5415.2009.02393.x 19682121

[R12] Buysse DJ, Reynolds IIICF, Monk TH, Berman SR, and Kupfer DJ (1989). The Pittsburgh Sleep Quality Index: a new instrument for psychiatric practice and research. Psychiatry Res. 28, 193–213. doi: 10.1016/0165-1781(89)90047-4 2748771

[R13] Campanini MZ, Mesas AE, Carnicero-Carreño JA, Rodríguez-Artalejo F, and Lopez-Garcia E (2019). Duration and quality of sleep and risk of physical function impairment and disability in older adults: results from the ENRICA and ELSA cohorts. Aging Dis. 10, 557–569. doi: 10.14336/AD.2018.0611 31165000 PMC6538215

[R14] Cappuccio FP, D’Elia L, Strazzullo P, and Miller MA (2010). Sleep duration and all-cause mortality: a systematic review and meta-analysis of prospective studies. Sleep 33, 585–592. doi: 10.1093/sleep/33.5.585 20469800 PMC2864873

[R15] Charlson ME, Pompei P, Ales KL, and MacKenzie CRA (1987). new method of classifying prognostic comorbidity in longitudinal studies: development and validation. J. Chronic Dis 40, 373–383. doi: 10.1016/0021-9681(87)90171-8 3558716

[R16] Chasens ER, Sereika SM, Weaver TE, and Umlauf MG (2007). Daytime sleepiness, exercise, and physical function in older adults. J. Sleep Res 16, 60–665. doi: 10.1111/j.1365-2869.2007.00576.x 17309764

[R17] Chennaoui M, Arnal PJ, Sauvet F, and Léger D (2014). Sleep and exercise: a reciprocal issue? Sleep Med. Rev 20, 59–72. doi: 10.1016/j.smrv.2014.06.008 25127157

[R18] Cole JC, Motivala SJ, Buysse DJ, Oxman MN, Levin MJ, Irwin MR, (2006). Validation of a 3-factor scoring model for the Pittsburgh sleep quality index in older adults. Sleep 29, 112–116. doi: 10.1093/sleep/29.1.112 16453989

[R19] Deer RR, Dickinson JM, Fisher SR, Ju H, and Volpi E (2016). Identifying effective and feasible interventions to accelerate functional recovery from hospitalization in older adults: A randomized controlled pilot trial. Contemp. Clin. Trials 49, 6–14. doi: 10.1016/j.cct.2016.05.001 27178766 PMC5351552

[R20] Deer RR, Goodlett SM, Fisher SR, Baillargeon J, Dickinson JM, Raji M, (2018). A randomized controlled pilot trial of interventions to improve functional recovery after hospitalization in older adults: feasibility and adherence. J. Gerontol. A Biol. Sci. Med. Sci 73, 187–193. doi: 10.1093/gerona/glx111 28591764 PMC5861905

[R21] Dew MA, Hoch CC, Buysse DJ, Monk TH, Begley AE, Houck PR, (2003). Healthy older adults’ sleep predicts all-cause mortality at 4 to 19 years of follow-up. Psychosomatic Med. 65, 63–73. doi: 10.1097/01.PSY.0000039756.23250.7C 12554816

[R22] Diem SJ, Ewing SK, Stone KL, Ancoli-Israel S, Redline S, Ensrud KE, (2014). Use of non-benzodiazepine sedative hypnotics and risk of falls in older men. J. Gerontol. Geriatr. Res 3, 158. doi: 10.4172/2167-7182.1000158 25587493 PMC4289612

[R23] English KL, Mettler JA, Ellison JB, Mamerow MM, Arentson-Lantz E, Pattarini JM, (2016). Leucine partially protects muscle mass and function during bed rest in middle-aged adults. Am. J. Clin. Nutr 103, 465–473. doi: 10.3945/ajcn.115.112359 26718415 PMC4733256

[R24] Foley D, Ancoli-Israel S, Britz P, and Walsh J (2004). Sleep disturbances and chronic disease in older adults: results of the 2003 national sleep foundation sleep in America survey. J. Psychosomatic Res 56, 497–502. doi: 10.1016/j.jpsychores.2004.02.010 15172205

[R25] Foley DJ, Vitiello MV, Bliwise DL, Ancoli-Israel S, Monjan AA, Walsh JK, (2007). Frequent napping is associated with excessive daytime sleepiness, depression, pain, and nocturia in older adults: findings from the National Sleep Foundation ‘2003 Sleep in America’ Poll. Am. J. Geriatric. Psychiatry 15, 344–350. doi: 10.1097/01.JGP.0000249385.50101.67 17384317

[R26] Fortier-Brochu É, and Morin CM (2014). Cognitive impairment in individuals with insomnia: clinical significance and correlates. Sleep 37, 1787–1798. doi: 10.5665/sleep.4172 25364074 PMC4196062

[R27] Fung CH, Martin JL, Chung C, Fiorentino L, Mitchell M, Josephson KR, (2012). Sleep disturbance among older adults in assisted living facilities. J. Am. Geriatr. Soc 20, 485–493. doi: 10.1097/JGP.0b013e318252e3e0 PMC3358504 22531104

[R28] Gooneratne NS, Weaver TE, Cater JR, Pack FM, Arner HM, Greenberg AS, (2003). Functional outcomes of excessive daytime sleepiness in older adults. J. Am. Geriatr. Soc 51, 642–649. doi: 10.1034/j.1600-0579.2003.00208.x 12752839

[R29] Greysen SR (2016). Activating hospitalized older patients to confront the epidemic of low mobility. JAMA Intern. Med 176, 928–929. doi: 10.1001/jamainternmed.2016.1874 27243416

[R30] Guralnik JM, Simonsick EM, Ferrucci L, Glynn RJ, Berkman LF, Blazer DG, (1994). A short physical performance battery assessing lower extremity function: association with self-reported disability and prediction of mortality and nursing home admission. J. Gerontol 49, M85–94. doi: 10.1093/geronj/49.2.M85 8126356

[R31] Hartley P, DeWitt AL, Forsyth F, Romero-Ortuno R, and Deaton C (2020). Predictors of physical activity in older adults early in an emergency hospital admission: a prospective cohort study. BMC Geriatr. 20, 177. doi: 10.1186/s12877-020-01562-3 32423418 PMC7236296

[R32] Hartz A, Bentler S, and Watson D (2003). Measuring fatigue severity in primary care patients. J. Psychosom. Res 54, 515–521. doi: 10.1016/S0022-3999(02)00600-1 12781305

[R33] Harvey AG, Stinson K, Whitaker KL, Moskovitz D, and Virk H (2008). The subjective meaning of sleep quality: a comparison of individuals with and without insomnia. Sleep 31, 383–393. doi: 10.1093/sleep/31.3.383 18363315 PMC2276747

[R34] Katz S (1983). Assessing self-maintenance: activities of daily living, mobility, and instrumental activities of daily living. J. Am. Geriatr. Soc 31, 721–727. doi: 10.1111/j.1532-5415.1983.tb03391.x 6418786

[R35] Keim SK, Ratcliffe SJ, Naylor MD, and Bowles KH (2020). Patient factors linked with return acute healthcare use in older adults by discharge disposition. J. Am. Geriatr. Soc 68, 2279–2287. doi: 10.1111/jgs.16645 33267559 PMC8352071

[R36] Kirkhus L, Harneshaug M, Šaltyte, Benth J, Grønberg BH, Rostoft S, Bergh S, (2019). Modifiable factors affecting older patients’ quality of life and physical function during cancer treatment. J. Geriatr. Oncol 10, 904–912. doi: 10.1016/j.jgo.2019.08.001 31444088

[R37] Kripke DF, Klauber MR, Wingard DL, Fell RL, Assmus JD, Garfinkel L, (1998). Mortality hazard associated with prescription hypnotics. Biol. Psychiatry 43, 687–693. doi: 10.1016/S0006-3223(97)00292-8 9583003

[R38] Lawton MP, and Brody EM (1969). Assessment of older people: self-maintaining and instrumental activities of daily living. Gerontologist 9, 179–186. doi: 10.1093/geront/9.3_Part_1.179 5349366

[R39] Manabe K, Matsui T, Yamaya M, Sato-Nakagawa T, Okamura N, Arai H, (2000). Sleep patterns and mortality among elderly patients in a geriatric hospital. Gerontology 46, 318–322. doi: 10.1159/000022184 11044786

[R40] Martin JL, Fiorentino L, Jouldjian S, Josephson KR, and Alessi CA (2010). Sleep quality in residents of assisted living facilities: effect on quality of life, functional status, and depression. J. Am. Geriatr. Soc 58, 829–836. doi: 10.1111/j.1532-5415.2010.02815.x 20722819 PMC3377484

[R41] Morisawa T, Saitoh M, Otsuka S, Takamura G, Tahara M, Ochi Y, (2021). Perioperative changes in physical performance affect short-term outcome in elderly cardiac surgery patients. Geriatr Gerontol Int. 21, 676–682. doi: 10.1111/ggi.14227 34212472

[R42] Olivier K, Gallagher RA, Killgore WDS, Carrazco N, Alfonso-Miller P, Gehrels J, (2016). Development and initial validation of the assessment of sleep environment: A novel inventory for describing and quantifying the impact of environmental factors on sleep. Sleep 39, A367. 10.3390/ijerph192013599 PMC9602531 36294179

[R43] Orff HJ, Drummond SP, Nowakowski S, and Perlis ML (2007). Discrepancy between subjective symptomatology and objective neuropsychological performance in insomnia. Sleep 30, 1205–1211. doi: 10.1093/sleep/30.9.1205 17910392 PMC1978394

[R44] Passos GS, Poyares D, Santana MG, D’Aurea CV, Youngstedt SD, Tufik S, (2011). Effects of moderate aerobic exercise training on chronic primary insomnia. Sleep Med. 12, 1018–1027. doi: 10.1016/j.sleep.2011.02.007 22019457

[R45] Perera S, Mody SH, Woodman RC, and Studenski SA (2006). Meaningful change and responsiveness in common physical performance measures in older adults. J. Am. Geriatr. Soc 54, 743–749. doi: 10.1111/j.1532-5415.2006.00701.x 16696738

[R46] Quality AfHR (2021). HCUP Fast Stats. Healthcare Cost and Utilization Project. Rockville, MD: Agency of Healthcare Research and Quality. 21413206

[R47] Reidy PT, Lindsay CC, McKenzie AI, Fry CS, Supiano MA, Marcus RL, (2018). Aging-related effects of bed rest followed by eccentric exercise rehabilitation on skeletal muscle macrophages and insulin sensitivity. Exp. Gerontol 107, 37–49. doi: 10.1016/j.exger.2017.07.001 28705613 PMC5762440

[R48] Rubio-Arias J, Marín-Cascales E, Ramos-Campo DJ, Hernandez AV, and Pérez-López FR (2017). Effect of exercise on sleep quality and insomnia in middle-aged women: A systematic review and meta-analysis of randomized controlled trials. Maturitas 100, 49–56. doi: 10.1016/j.maturitas.2017.04.003 28539176

[R49] Saitoh M, Takahashi Y, Okamura D, Akiho M, Suzuki H, Noguchi N, (2021). Prognostic impact of hospital-acquired disability in elderly patients with heart failure. ESC Heart Fail. 8, 1767–1774. doi: 10.1002/ehf2.13356 33838022 PMC8120367

[R50] Salpakoski A, Törmäkangas T, Edgren J, Sihvonen S, Pekkonen M, Heinonen A, (2014). Walking recovery after a hip fracture: a prospective follow-up study among community-dwelling over 60-year old men and women. Biomed. Res. Int 2014, 289549. doi: 10.1155/2014/289549 24511530 PMC3912885

[R51] Soehner AM, and Harvey AG (2012). Prevalence and functional consequences of severe insomnia symptoms in mood and anxiety disorders: results from a nationally representative sample. Sleep 35, 1367–1375. doi: 10.5665/sleep.2116 23024435 PMC3443763

[R52] Stone KL, Blackwell TL, Ancoli-Israel S, Cauley JA, Redline S, Marshall LM, (2014). Sleep disturbances and risk of falls in older community-dwelling men: the outcomes of sleep disorders in older men (MrOS Sleep) study. J. Am. Geriatr. Soc 62, 299–305. doi: 10.1111/jgs.12649 24428306 PMC3945231

[R53] Su X, and Wang DX (2018). Improve postoperative sleep: what can we do? Curr. Opin. Anaesthesiol 31, 83–88. doi: 10.1097/ACO.0000000000000538 29120927 PMC5768217

[R54] Tanner RE, Brunker LB, Agergaard J, Barrows KM, Briggs RA, Kwon OS, (2015). Age-related differences in lean mass, protein synthesis and skeletal muscle markers of proteolysis after bed rest and exercise rehabilitation. J. Physiol 593, 4259–4273. doi: 10.1113/JP270699 26173027 PMC4594296

[R55] Vitale KC, Owens R, Hopkins SR, and Malhotra A (2019). Sleep hygiene for optimizing recovery in athletes: review and recommendations. Int, J, Sports Med. 40, 535–543. doi: 10.1055/a-0905-3103 31288293 PMC6988893

[R56] Wang M, Tan Y, Shi Y, Wang X, Liao Z, Wei P, (2020). Diabetes and sarcopenic obesity: pathogenesis, diagnosis, and treatments. Front. Endocrinol 11, 568. doi: 10.3389/fendo.2020.00568 PMC7477770 32982969

[R57] Weiss A, and Elixhauser A (2014). Overview of Hospital Stays in the United States, 2012 Healthcare Cost and Utilization Project. Rockville, MD: Agency for Healthcare Research and Quality. 25506966

[R58] Yilmaz D, Tanrikulu F, and Dikmen Y (2017). Research on sleep quality and the factors affecting the sleep quality of the nursing students. Curr Health Sci J. 43, 20–24. doi: 10.12865/CHSJ.43.01.03 30595850 PMC6286721

[R59] Yuen EJ, Zisselman MH, Louis DZ, and Rovner BW (1997). Sedative-hypnotic use by the elderly: effects on hospital length of stay and costs. J. Mental. Health Administ 24, 90–97. doi: 10.1007/BF02790484 9033160

[R60] Zisselman MH, Rovner BW, Yuen EJ, and Louis DZ (1996). Sedative-hypnotic use and increased hospital stay and costs in older people. J. Am. Geriatr. Soc 44, 1371–1374. doi: 10.1111/j.1532-5415.1996.tb01410.x 8909355

