# Supplementary material for: Improvements in sleep quality and fatigue are associated with improvements in functional recovery following hospitalization in older adults
Source: Front Sleep. 2022 Oct 14;1:1011930. doi: 10.3389/frsle.2022.1011930 (PMC10217784; doi:10.3389/frsle.2022.1011930)
Supplement: Supplementary file 1 [file Data_Sheet_1.docx]

| **Modified Assessment of Sleep Environment (SEQ):** | **Yes** | **No** |
| --- | --- | --- |
| My sleep differed during my time at UTMB hospital than my usual sleep patterns at home |  |  |
| The room was so warm or cold that it disturbed my sleep |  |  |
| Light from the window or other source disturbed my sleep |  |  |
| The mattress or pillow(s) were uncomfortable and disturbed my sleep |  |  |
| During the night, I could hear noise outside the room that disturbed my sleep |  |  |
| Noise inside the room (from the air vents, pipes, etc.,) was so loud that I was aware of the sounds and it disturbed my sleep |  |  |
| The concern and support of staff provided me a level of comfort such that I rarely if ever worried about being safe at night |  |  |
| I engaged in any behaviors during a time I would normally be asleep at home (e.g., watched TV, read, worried, worked, checked email/internet) |  |  |
| It was uncomfortable (or caused me pain) being confined to bed and this disturbs my sleep |  |  |
| Other things related to being at the UTMB hospital disturbed my sleep |  |  |

**Supplemental Figure 1**: The modified version of the sleep environment questionnaire (SEQ) used for this present study. The original questionnaire was modified to be used to collect quality improvement data for improving the hospital sleep environment.


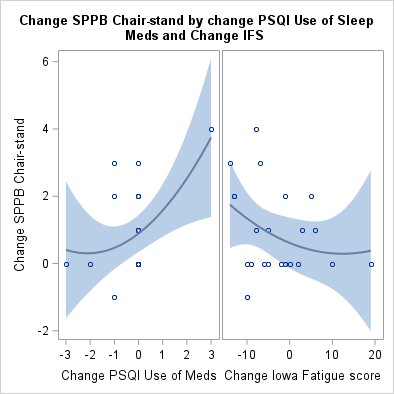

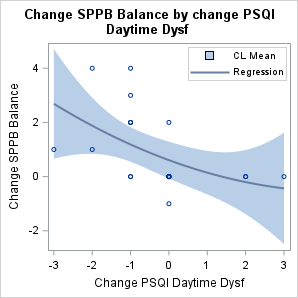

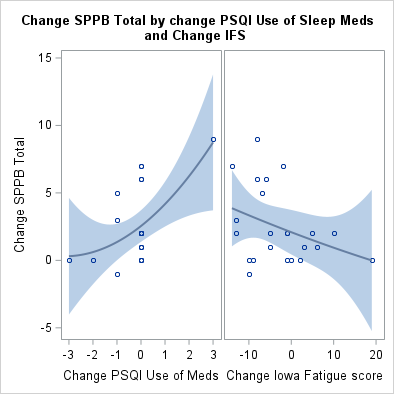

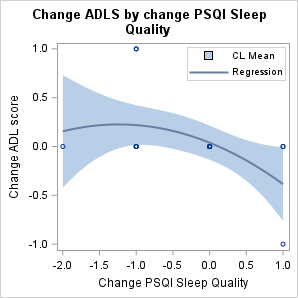

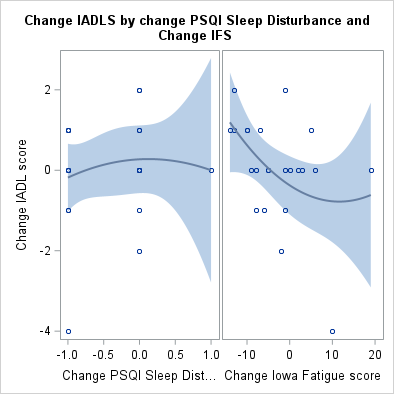


**A.**

**B.**

**C.**

**D.**

**E.**

**Supplemental Figure 2:** Generalized regression plots of significant associations between changes in sleep and physical function outcomes in older hospitalized patients from an in-hospital baseline assessment to 4 weeks post-hospital discharge. The plots depict (A) with increase change PSQI Sleep Quality (worse sleep), there is significant decrease in change ADLS (less independence), p=0.03; (B) with increase change PSQI day time Dysfunction (worse sleep), there is significant decrease in change SPPB Balance (worse physical performance recovery), p=0.02; (C) with increase change PSQI use of sleep medications (more medication), there is significant increase in change SPPB Total (better physical performance recovery), p=0.001 and with increase change Iowa fatigue score (more fatigue), there is significant decrease in change SPPB Total (worse physical performance recovery), p=0.01; (D) with increase change PSQI use of sleep medications (more medication), there is significant increase in change SPPB Chair-stand (better physical performance recovery), p=0.01 and with increase change Iowa fatigue score (more fatigue), there is significant decrease in change SPPB Chair-Stand (worse physical performance recovery), p=0.03 and (E) with increase change PSQI Sleep Disturbance (worse sleep), there is significant decrease in change IADLS (less independence), p=0.03 and with increase change Iowa fatigue score (more fatigue), there is significant decrease in change IADLS (less independence), p=0.005.
