# Supplementary material for: Improvements in sleep quality and fatigue are associated with improvements in functional recovery following hospitalization in older adults
Source: Front Sleep. 2022 Oct 14;1:1011930. doi: 10.3389/frsle.2022.1011930 (PMC10217784; doi:10.3389/frsle.2022.1011930)
Supplement: Supplementary file 2 [file Table_1.DOCX]

**Supplemental Table 1:** Regression analysis between baseline (in-hospital) sleep and physical function outcomes^1^

|  | SPPB Total | SPPB Balance | SPPB Chair Stand | SPPB Gait | ADL | IADL |
| --- | --- | --- | --- | --- | --- | --- |
| PSQI Global | -0.14(0.21) | -0.05(0.07) | -0.01(0.08) | -0.08(0.07) | 0.01(0.06) | 0.04(0.09) |
| PSQI Sleep Quality | 4.07(2.97) | 1.31(0.99) | 1.36(1.21) | 1.40(1.02) | -0.73(0.72) | 1.26(1.24) |
| PSQI Sleep latency | -1.29(1.67) | -0.37(0.56) | -0.31(0.68) | -0.63(0.58) | 0.15(0.41) | -0.08(0.70) |
| PSQI Sleep Duration | -1.42(1.80) | -0.54(0.60) | -0.36(0.73) | -0.52(0.62) | -0.24(0.44) | -0.92(0.75) |
| PSQI Sleep Efficiency | 0.80(1.87) | 0.81(0.63) | -0.19(0.76) | 0.18(0.65) | -0.22(0.46) | 0.44(0.78) |
| PSQI Sleep Disturbance | -1.47(2.32) | -0.95(0.78) | 0.22(0.94) | -0.75(0.80) | 1.11(0.56) | -0.55(0.97) |
| PSQI Use of Meds | 0.71(1.01) | 0.07(0.34) | 0.27(0.41) | 0.37(0.35) | -0.26(0.25) | 0.34(0.42) |
| PSQI Daytime Dysfunction | 0.21(1.65) | -0.21(0.55) | 0.12(0.67) | 0.29(0.57) | 0.16(0.40) | 0.57(0.69) |
| IFS | -0.43(0.34) | -0.08(0.11) | -0.17(0.14) | -0.17(0.12) | 0.07(0.08) | -0.14(0.14) |

^1^Values are represented as β (SE). β=regression coefficient (represents the slope of the linear relation of the predictor variable and the outcome variable), SE = standard error.

PSQI = Pittsburgh Sleep Quality Index; IFS=Iowa Fatigue Score; SPPB = Short Physical Performance Battery; ADL = Activities of Daily Living Score; IADL = Instrumental Activities of Daily Living Score
