# Supplementary material for: Improvements in sleep quality and fatigue are associated with improvements in functional recovery following hospitalization in older adults
Source: Front Sleep. Author manuscript; Available in PMC 2023 May 26. (PMC10217784; doi:10.3389/frsle.2022.1011930)
Supplement: Supplementary file 1 [file NIHMS1899367-supplement-Supplementary_Figure_1.docx]

**Original Assessment of Sleep Environment:**					**Modified Assessment of Sleep Environment (SEQ):**

|  | **Yes** | **No** |
| --- | --- | --- |
| My sleep differed during my time at UTMB hospital than my usual sleep patterns at home |  |  |
| The room was so warm or cold that it disturbed my sleep |  |  |
| Light from the window or other source disturbed my sleep |  |  |
| The mattress or pillow(s) were uncomfortable and disturbed my sleep |  |  |
| During the night, I could hear noise outside the room that disturbed my sleep |  |  |
| Noise inside the room (from the air vents, pipes, etc.,) was so loud that I was aware of the sounds and it disturbed my sleep |  |  |
| The concern and support of staff provided me a level of comfort such that I rarely if ever worried about being safe at night |  |  |
| I engaged in any behaviors during a time I would normally be asleep at home (e.g., watched TV, read, worried, worked, checked email/internet) |  |  |
| It was uncomfortable (or caused me pain) being confined to bed and this disturbs my sleep |  |  |
| Other things related to being at the UTMB hospital disturbed my sleep |  |  |

I have difficulty sleeping because the place where I sleep...

|  | Strongly Agree | Agree | Disagree | Strongly Disagree |
| --- | --- | --- | --- | --- |
| Has too much light |  |  |  |  |
| Is too dark |  |  |  |  |
| Is too noisy |  |  |  |  |
| Is too quiet |  |  |  |  |
| Is too warm |  |  |  |  |
| Is too cool |  |  |  |  |
| Is too humid |  |  |  |  |
| Has an uncomfortable smell |  |  |  |  |
| Has uncomfortable pillows or blankets |  |  |  |  |
| Has a mattress or other sleeping surface that is too firm |  |  |  |  |
| Has a mattress or other sleeping surface that is too soft |  |  |  |  |
| Has a mattress or other sleeping surface that is uncomfortable for another reason |  |  |  |  |
| Does not feel safe and secure |  |  |  |  |

**Supplemental Figure 1**: The original sleep environment questionnaire and the modified version of the sleep environment questionnaire (SEQ) used for this present study. The original questionnaire was modified to be used to collect quality improvement data for improving the hospital sleep environment.
