# Supplementary material for: Improvements in sleep quality and fatigue are associated with improvements in functional recovery following hospitalization in older adults
Source: Front Sleep. Author manuscript; Available in PMC 2023 May 26. (PMC10217784; doi:10.3389/frsle.2022.1011930)
Supplement: Supplementary file 2 [file NIHMS1899367-supplement-Supplementary_Figure_2.docx]

**A.**

**B.**

**C.**

**D.**

**E.**

**Supplemental Figure 2:** Generalized regression plots of significant associations between changes in sleep and physical function outcomes in older hospitalized patients from an in-hospital baseline assessment to 4 weeks post-hospital discharge. The plots depict (A) with increase change PSQI Sleep Quality (worse sleep), there is significant decrease in change ADLS (less independence), p=0.03; (B) with increase change PSQI day time Dysfunction (worse sleep), there is significant decrease in change SPPB Balance (worse physical performance recovery), p=0.02; (C) with increase change PSQI use of sleep medications (more medication), there is significant increase in change SPPB Total (better physical performance recovery), p=0.001 and with increase change Iowa fatigue score (more fatigue), there is significant decrease in change SPPB Total (worse physical performance recovery), p=0.01; (D) with increase change PSQI use of sleep medications (more medication), there is significant increase in change SPPB Chair-stand (better physical performance recovery), p=0.01 and with increase change Iowa fatigue score (more fatigue), there is significant decrease in change SPPB Chair-Stand (worse physical performance recovery), p=0.03 and (E) with increase change PSQI Sleep Disturbance (worse sleep), there is significant decrease in change IADLS (less independence), p=0.03 and with increase change Iowa fatigue score (more fatigue), there is significant decrease in change IADLS (less independence), p=0.005.
